# Seroprevalence against *Borrelia burgdorferi* sensu lato and occurence of antibody co-expression with *Anaplasma phagocytophilum* in dogs in Latvia

**DOI:** 10.1186/2046-0481-66-9

**Published:** 2013-05-21

**Authors:** Inese Berzina, Ilze Matise

**Affiliations:** 1 Latvia University of Agriculture, Faculty of Veterinary Medicine Preclinical Institute, Pathology department, Kr. Helmana Street 8, Jelgava, LV-3004, Latvia

**Keywords:** *B. burgdorferi* sensu lato, *A. phagocytophilum*, Dogs, Seroprevalence, Latvia

## Abstract

**Background:**

Lyme disease is commonly diagnosed in humans in Latvia, but up to date no studies have been performed to investigate its prevalence in dogs. The aim of this study was to evaluate if seroprevalence against *B. burgdorferi* sensu lato (*B. burgdorferi* s.l.) and co-expression of antibodies against *B.burgdorferi* s.l. and *A. phagocytophilum* is higher in dogs with clinical suspicion of tick-borne diseases compared to healthy dogs.

**Findings:**

Venous blood was taken from healthy dogs (n=441) and dogs suspected to have borreliosis and/ or canine granulocytic anaplasmosis (n=29). The presence of antibodies was detected with SNAP 4Dx test (IDEXX, Westbrook, Maine, USA). The seroprevalence against *B. burgdorferi* s.l. in healthy dogs was 2.49% (11/441) and 36% (4/11) of seropositive dogs had antibodies against both of investigated bacteria. None of the dogs in sick dog group had detectable antibodies against *B. burgdorferi* s.l.

**Conclusions:**

We conclude that seroprevalence to *B. burgdorferi* s.l. in dogs in Latvia is low and that dogs with suspicion of tick-borne disease do not have higher *B. burgdorferi* s.l. seroprevalence than healthy dogs. Dogs that express antibodies against *B. burgdorferi* s.l. frequently co-express antibodies against *A. phagocytophilum.*

## Findings

Lyme disease and granulocytic anaplasmosis have been diagnosed in humans in Latvia (Bormane, [1]; Süss, [2]). *Ixodes ricinus* and *I. persulcatus* ticks in Latvia carry several infectious agents, including *Borrelia burgdorferi* sensu lato (s.l.) and *A. phagocytophilum* (Ranka et al., [3]; Bormane, [1]). The seroprevalence against *Anaplasma phagocytophilum* in healthy dogs in Latvia is reported to be 11% and recently a canine granulocytic anaplasmosis (CGA) case was described (Berzina et al., [4]). Dogs with borreliosis caused by *B. burgdorferi* and CGA caused by *A. phagocytophilum* present with nonspecific clinical signs – lethargy, inappetence, fever, lameness (Beall et al., [5]). Most of the naturally infected dogs resolve these infections without showing clinical signs, however, in cases of double infections disease can be more severe and response to the treatment can be weaker or take longer (Beall et al., [5]). Dogs are recognized as sentinel for human tick-borne diseases, therefore locally generated information on the seroprevalence may be useful for veterinarians and human health specialists alike (Goossens et al., [6]; Hamer et al., [7]).

The aim of this study was to evaluate if seroprevalence against *B. burgdorferi* s.l. and co-expression of antibodies against *B.burgdorferi* s.l. and *A. phagocytophilum* is higher in dogs with clinical suspicion of tick-borne diseases compared to clinicaly healthy dogs.

Two groups of dogs were evaluated – clinically healthy dogs (further in the text - healthy dogs, n= 441) and dogs suspected to have borreliosis and/ or CGA (further in the text – sick dogs, n=29). The sampling of healthy dogs was planned to cover urban and rural locations and 3 regions depending on the prevalent tick species (*I. ricinus* (IR region, n=272), *I. persulcatus* (IP region, n=93), mixed region (M region, n=76). The number of collected samples was roughly proportional to the density of human population. Detailed explanation on the sample collection and inclusion criteria of dogs have been published previously (Berzina et al., [4]). Two milliliters of venous blood was taken from dogs and serological testing was performed (SNAP 4Dx test, IDEXX Laboratories, Westbrook, Maine, USA) with test that detects antibodies against *A. phagocytophilum*, *B. burgdorferi* sensu lato, *Ehrlichia canis* and antigen of *Dirofilaria immitis*. Statistical analysis of the data consisted of the calculation of descriptive statistics and seroprevalence against the pathogens in each of the dog groups (NCSS, 2007, Utah, USA).

In healthy dogs seroprevalence against *B. burgdorferi* s.l. was 2.49% (11/441). More than one third 36% (4/11) of seropositive dogs co-expressed antibodies against *B. burgdorferi* s.l. and *A. phagocytophilum*. None of the dogs in the sick dog group expressed antibodies against *B. burgdorferi* s.l. Seven female and four male dogs, from eight breeds and 1 unknown breed dog were seropositive to *B. burgdorferi*. We did not find significant differences in age, sex or breeds of dogs seropositive against *A. phagocytophilum* versus those seropositive against *B. burgdorferi* s.l. (Berzina et al., [4]).

Co-expression of antibodies was detected only in healthy pet dogs. Three female and one male dog from four different breeds were seropositive against both *B. burgdorferi* s.l. and *A. phagocytophilum*. No statistically significant difference (p=0.62) was found between the mean ages of the dogs seropositive against *B. burgdorferi* s.l. (5.7 years, s= 2.9) and double seropositive dogs (6.7 years, s=3.3).

**Figure 1:**
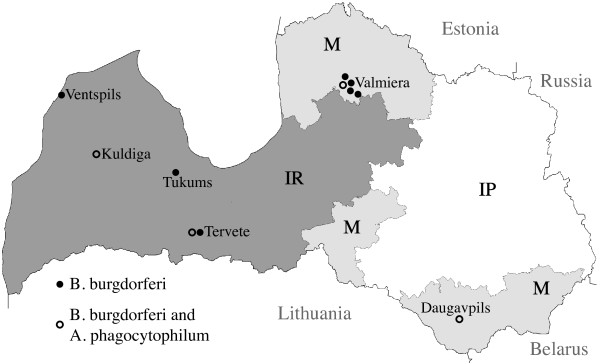
**Distribution of dogs seropositive against *B. burgdorferi *sensu lato (*B. burgdorferi *s.l.) and dogs seropositive against *A. phagocytophilum *and *B. burgdorferi *s.l. IR - *I. ricinus *tick habitat, IP - *I. persulcatus *tick habitat, M – both tick species have been detected.**

Geographical distribution of the seropositive and double seropositive dogs is displayed in the Figure 1. Overall, we can see the trend of higher seropositivity in IR and M regions. Similarly the seropositivity against *A. phagocytophilum* was significantly higher in dogs from IR and M regions (Berzina et al., [4]). However, study by Bormane [1] shows that in Latvia borrelial DNA was equally isolated from *I. ricinus and I. persulcatus*. Both of these tick species have been described as main vectors associated with Lyme disease, *I. ricinus* in Northern Europe and *I. persulcatus* in the Eastern Europe (Gray, [8]). To our knowledge, no studies have been conducted to evaluate if these tick species in Latvia have different preferences on feeding on dogs or humans. Additional information on this issue might be added by our ongoing molecular study on the ticks collected from dogs living in IR, IP and M regions of Latvia.

Low seropositivity against borrelia in healthy and lack of seropositivity in sick dogs was unexpected, given the rather high percentage of ticks carrying borrelia and the known incidence of Lyme disease of 31.8 cases per 100,000 humans in Latvia (Ranka et al., [3]; Halperin, [9]). Reported seroprevalence for *B. burgdorferi* s.l. in dogs in Europe is similarly low 3.9% in Sweden, 6.5% in Czech Republic, 1.09% in France (Egenvall et al., [10]; Pejchalovà et al., [11]; Pantchev et al., [12]) while human disease is common, reaching 69 cases per 100,000 inhabitants in Sweden (Ornastein et al., [13]).

Several authors describe low seropositivity in dogs and even lower number of clinical cases (Beall et al., [5]; Pantchev et al., [12]). This can be partially explained by particularities of the transmission of borrelia to the mammalian host. Transmission can be affected by several factors that include the concentration of the bacteria, length of the feeding of the tick and pathogen species (Kidd et al., [14]). Our results might be affected by several factors –low number of dogs in sick dog group as well as possibility that these dogs might have received antibiotics prior to our testing; the latter might suppress the antibody titer below the detection level of the test (Savic, [15]). Additionally, our results might be affected also by the lower number of investigated dogs from IP and M regions.

Serological test for *B. burgdorferi* s.l. used in this study is sensitive and specific (94% and 99.5% respectively) and detects C6 protein of *B. burgdorferi* s.l. that is specific to the natural infection, thus vaccinated dogs would be excluded (Beall et al., [5]; Pantchev et al., [12]; Couto et al., [16]).

Although in this study we detected only *B. burgdorferi* s.l. and *A. phagocytophilum* antibody co-expression, the possibility of other diseases that are not typical for this geographical location (e.g. ehrlichiosis) still should be kept in mind since pathogens can adapt to be transmitted by a new tick species or ticks can widen their area of habitat or can be “imported” in the country by travelling animals or migrating birds (Cieniuch et al., [17]; Skotarczak et al., [18]; Beall, et al., [5]; Rand et al., [19]).

We conclude that seroprevalence against *B. burgdorferi* s.l. in dogs in Latvia is low, but seropositive dogs frequently co-express antibodies against *A. phagocytophilum*.

## Abbreviations

B. burgdorferi s.l: *B. burgdorferi* sensu lato; CGA: Canine granulocytic anaplasmosis; IR region: Region of Latvia, where *I. ricinus* ticks prevail; IP region: Region of Latvia where *I. persulcatus* ticks prevail; M region: Region of Latvia where *I. ricinus* and *I.persulcatus* ticks have been found.

## Competing interests

The authors declare that they have no competing interests.

## Authors’ contributions

IB performed the sampling, analysis and wrote the draft of this report. IM supervised all stages of the work presented in this report and critically read the report. Both authors read and approved the final manuscript.
